# Photocatalytic Investigation of Aerosol-Assisted Atmospheric Pressure Plasma Deposited Hybrid TiO_2_ Containing Nanocomposite Coatings

**DOI:** 10.3390/nano12213758

**Published:** 2022-10-26

**Authors:** Chiara Lo Porto, Massimo Dell’Edera, Ilaria De Pasquale, Antonella Milella, Francesco Fracassi, Maria Lucia Curri, Roberto Comparelli, Fabio Palumbo

**Affiliations:** 1 CNR-IPCF, Istituto per i Processi Chimico-Fisici, S.S. Bari, c/o Dip. Chimica Via Orabona 4, 70126 Bari, Italy; 2 Dipartimento di Chimica, Università degli Studi di Bari Aldo Moro, Via Orabona 4, 70126 Bari, Italy; 3 CNR-NANOTEC, c/o Dip. Chimica Via Orabona 4, 70126 Bari, Italy

**Keywords:** plasma deposition, nanocomposite coating, TiO_2_, photocatalysis, aerosol-assisted plasma

## Abstract

We report on the aerosol-assisted atmospheric-pressure plasma deposition onto a stainless-steel woven mesh of a thin nanocomposite coating based on TiO_2_ nanoparticles hosted in a hybrid organic–inorganic matrix, starting from nanoparticles dispersed in a mixture of hexamethyldisiloxane and isopropyl alcohol. The stainless-steel mesh was selected as an effective support for the possible future technological application of the coating for photocatalytically assisted water depollution. The prepared coatings were thoroughly investigated from the chemical and morphological points of view and were demonstrated to be photocatalytically active in the degradation of an organic molecule, used as a pollutant model, in water upon UV light irradiation. In order to optimize the photocatalytic performance, different approaches were investigated for the coating’s realization, namely (i) the control of the deposition time and (ii) the application of a postdeposition O_2_ plasma treatment on the pristine coatings. Both strategies were found to be able to increase the photocatalytic activity, and, remarkably, their combination resulted in a further enhancement of the photoactivity. Indeed, the proposed combined approach allowed a three-fold increase in the kinetic constant of the degradation reaction of the model dye methylene blue with respect to the pristine coating. Interestingly, the chemical and morphological characterizations of all the prepared coatings were able to account for the enhancement of the photocatalytic performance. Indeed, the presence of the TiO_2_ nanoparticles on the outmost surface of the film confirmed the accessibility of the photocatalytic sites in the nanocomposite and reasonably explained the enhanced photocatalytic performance. In addition, the sustained photoactivity (>5 cycles of use) of the nanocomposites was demonstrated.

## 1. Introduction

Technological advances in many human activities in the last decades are reflected in the severe modification of the environment, including ever-increasing water pollution. The anthroposphere-generated pollutants include a variety of heavy metals and organic molecules, including the so-called emerging pollutants. This last class of components, under increasing observation in recent times, comprises persistent organic pollutants (POPs), pharmaceuticals and personal care products (PPCPs), endocrine-disrupting chemicals (EDCs), and agricultural chemicals (pesticides and herbicides). Moreover, several pathogenic microorganisms contribute to water pollution, as they can grow and proliferate therein, spreading infections [1,2]_._

Several methods have been developed and many more are currently being explored for water remediation. One of the most-investigated classes of approach relies on advanced oxidation processes (AOPs) that mainly involve the generation of hydroxyl radicals (OH·) [3]. Among the AOPs, photocatalytic processes are based on the use of a photocatalyst generating, upon UV irradiation in presence of water or humidity, oxygen radicals that are able to attack and degrade organic molecules, thus reducing the concentrations of pollutants in the water [4,5,6,7,8,9,10,11]. Titanium dioxide, TiO_2_, is one of the most-used photocatalytic materials for environmental applications due to its high chemical stability, high efficiency, and cost-effectiveness, while its toxicity, previously considered low, has recently been re-evaluated, and TiO_2_ is now banned as a food additive according to the European regulation [12]. TiO_2_ nanoparticles (NPs) possess a photocatalytic activity higher than their bulk counterpart due to the much higher surface area available for photocatalysis [13,14,15,16,17,18]. However, the applications of photocatalytic NPs, and specifically those addressing water depollution via AOPs, requires the careful consideration of the technological and safety issues concerning their recovery from water bodies. To tackle the critical issue of NP dispersion in the environment and the related hazard for human and/or environmental health [12] and to circumvent the evident complexity of NP recovery and the scarce viability of filtration processes for this purpose, a range of procedures have been proposed for NP deposition onto supports suited for their integration in water treatment plants, taking advantage of both bottom-up and top-down coating fabrication processes [19]. Indeed, the immobilization of a nanostructured photocatalyst onto a substrate suitable to be integrated into systems and devices, such as photoreactors to be included in treatment processes, represents a more feasible and sustainable solution to prevent dispersion into the environment. This class of strategies results in particularly amenable routes for integration in depollution units for water treatment, though they suffer from an unavoidable trade-off in terms of surface availability and hence photocatalytic performance due to the reduction in the amount of surface sites available for photocatalytic processes [20,21,22]. Among the several approaches reported for the immobilization of photocatalytic nanomaterials, the design and fabrication of a coating formed of a nanocomposite embedding the photocatalytic NPs in a polymeric host matrix represents a very effective and technologically viable procedure that is able, in principle, to combine the characteristics of the host matrix in terms of mechanical, chemical, and processability properties with the peculiar size-dependent properties of the nanofillers [23]. Indeed, a wide range of techniques is currently available for conformally depositing a film of photocatalytic nanocomposite onto different solid substrates and addressing specific applications. Among techniques that include sol–gel, hydrothermal, chemical, and physical vapor deposition and dip coating [19], plasma deposition methods, in particular those relying on the use of aerosol-assisted atmospheric pressure (AAAP) plasma, are particularly advantageous, as they can be successfully applied to substrates with different compositions and geometries, are flexible, feature 3D textures, and are characterized by low resistance to high temperatures and pressures. In addition, these methods are extremely versatile, as they allow the composition of the host matrix as well as the NP fillers to be varied easily by simply tuning the deposition parameters, making them suitable for the realization of coatings for diverse application fields [22].

AAAP plasma deposition is based on the use of a precursor for the matrix, which can be a gas, which could be directly released into the reaction chamber, or a liquid, and, in this case, a preliminary spraying step would be required for it to be released into the chamber as an aerosol.

In NP-based AAAP plasma nanocomposite deposition, NPs are suspended in a proper solvent or directly into the liquid precursor. In both cases, the suspension is sprayed into the plasma chamber [24,25]. The AAAP plasma deposition has often been reported as an effective route to obtain nanocomposite coatings based on inorganic NPs [26,27,28,29].

However, to the best of our knowledge, only a few of these reports accounted for the actual photocatalytic performance of the AAAP-plasma-deposited nanocomposite films [30]. Indeed, Uricchio et al. [30] proposed a coating obtained by spraying an aerosol of an oleate-capped TiO_2_ suspension in n-octane onto a substrate formed of polyurethane foam, thus providing not only a clear indication of the suitability of the methods for photocatalytic film deposition but also proving its photocatalytic activity in a simulated sample.

Here, we report on the AAAP plasma deposition of a nanocomposite coating embedding uncapped commercial TiO_2_ NPs into a hybrid organosilane matrix obtained by feeding the plasma with a hexamethyldisiloxane/iso-propylalcohol (HMDSO/IPA) mixture onto a metallic mesh. The composition of the feeding mixture was properly defined in order to generate a hybrid host matrix that was particularly suited to be less sensitive to the photochemical degradation assisted by TiO_2_ nanoparticles. In addition, the woven stainless-steel mesh was selected as an unconventional substrate, as it offers a large surface for the deposition of the nanocomposite coating, which contributes to compensating for the loss of surface area due to NP immobilization and, at the same time, is technologically well-suited for its future integration into water treatment photoreactors, which is essential to a real-scale application of this class of AOP.

The effects of deposition time and postdeposition O_2_ plasma treatment on the structure and morphology of the obtained nanocomposite coatings were investigated, and their photocatalytic behaviors were evaluated by monitoring the discoloration, under UV light exposure, of an organic dye, methylene blue, in aqueous solution as a target model organic molecule of the photocatalytic process. All the prepared coatings were thoroughly characterized, and the observed highly photocatalytic performances are discussed on the basis of the structure and the morphology of the nanocomposite films. The investigation demonstrated the enhanced performance of the coatings obtained with longer deposition times and assessed the effect of postdeposition O_2_ plasma treatment. The photocatalytic behavior was found to be sustained after several cycles of usage.

The proposed approach to photocatalytic nanocomposite deposition represents a valuable advance towards their its application in water treatment, thus holding great promise for its usage as a scalable integration in a suitably designed plant.

## 2. Materials and Methods

### 2.1. Materials

TiO_2_ P25 Evonik was used as photocatalytic nanomaterial (10–50 nm nanoparticle diameter, as reported in the Supplementary Materials, Figure S1). In these experiments, 710 μm-thick chips (1 × 1 cm^2^) of double-face polished crystalline silicon (100) (MicroChemicals GmbH, Ulm, Gemany) and stainless-steel meshes (LPS Tele INOX 316L, Monza, Italy) were used as substrates, respectively, for the chemical, morphological, and structural characterization of the nanocomposite films (XPS, FT-IR spectroscopy, SEM and profilometry) and for the chemical and morphological (SEM/EDX) investigation and for the evaluation of their photocatalytic activity.

Hexamethyldisiloxane ≥ 98% (HMDSO, Sigma Aldrich, Darmstadt, Germany) and isopropyl alcohol ≥ 99.8% (IPA, Honeywell, Charlotte, NC, USA) were used to prepare the dispersion used as an aerosol feed. He (99.999%) (Air Liquide, Milano, Italy) was used as a gas feed for the plasma deposition process. Methylene blue (3,7-bis(Dimethylamino)-phenazathionium chloride, MB) purchased from Aldrich Chemical reagents and Milli-Q quality water (Millipore, Bedford, MA, USA) were used for the photocatalytic activity evaluation.

### 2.2. Plasma Deposition and Treatment of Coatings

An in-house-built dielectric barrier discharge (DBD) reactor, featuring specifications described in detail elsewhere and outlined in the Supplementary Materials (Figure S2), was used [31,32]. Briefly, the reactor consisted of two parallel plates of silver-coated alumina electrodes (5 × 8 cm^2^ wide and 0.63 mm thick) separated by a 3 mm gap. The electrodes were confined in a Plexiglas chamber, and the plasma discharge was ignited between the electrodes while the sample to be coated was placed onto the bottom one. The aerosol was generated by releasing a 4 slm flow of He in a pneumatic atomizer (mod. 3076, TSI). The liquid dispersion feeding the aerosol was formed of a suspension of TiO_2_ (10 mg/mL) in HMDSO/IPA (10/90 *v*/*v*). Before the film deposition, the suspension was ultrasonicated for 10 min, and it was kept under constant stirring during the process. An additional 4 slm of He was supplied to the discharge. The gas flow rate was controlled by electronic mass flow controllers (MSK Instruments, Reggio Emilia, Italy). The gas/aerosol feed was released into the reaction chamber by means of a slit, passed through the plasma zone, and was finally pumped out by an aspirator located on the opposite site of the chamber. The discharge was ignited in continuous mode using a wideband AC power amplifier (Al-1000-HF-A by AMP-LINE corp.) driven by a function generator (Model TG-1000 by TTi) The amplifier was connected to the high-voltage electrode by a HV transformer (Model AL-T1000, AMP-LINE corp.). The electrical characteristics of the plasma were investigated, measuring the voltage and the current delivered to the system with a high-voltage (P6015A, Tektronix, Beaverton, Oregon, USA) probe and a resistance-type current probe, both connected to an oscilloscope (TDS 2014C, Tektronix, Beaverton, Oregon, USA). A power of 1.67 W/cm^2^ was applied to generate plasma with a peak-to-peak voltage of 5 kV and a frequency of 24 kHz. Two types of coatings were obtained as a function of the deposition time, namely the *PD3m* sample (*PD* stands for *plasma deposited*) for 3 min and the *PD20m* sample for 20 min. Every discharge was preceded and followed by a 5 min purging step with 4 slm of He.

The postdeposition plasma treatment was carried out in an in-house-built parallel-plate capacitive plasma reactor operated at low pressure, as reported and illustrated in the Supplementary Materials (Figure S3) [33]. All the deposited coatings were treated by feeding the plasma with 20 sccm of O_2_ at 300 mTorr with 80 W of RF power for 5 min. The postdeposition plasma-treated coatings are referred to as the *PD3m-P* and *PD20m-P* samples.

### 2.3. Characterization of the Coatings

The thickness of the coatings was evaluated by means of a KLA-Tencor (Milpitas, CA, USA) D-120 profilometer using polished silicon chips as substrates and scratching part of the coating with a scalpel.

The morphology of the deposited films was investigated by a field emission gun—scanning electron microscopy (FEG-SEM) analysis carried out with a Zeiss Supra 40 SEM equipped with a Gemini field-effect emission gun. The extraction voltage was set to 3 kV, and the brightness, contrast, and working distance (varying in the range of 2–4 mm) were optimized for each acquisition.

Energy-dispersive spectroscopy (EDS) was used to study the chemical composition of the coating surface and was carried out using an INCA Oxford microanalysis probe mounted onto the Zeiss Supra 40 SEM. The results are reported as Ti/Fe in order to highlight the variation in Ti on the mesh, as iron was present in the mesh composition, and as the Ti/Si ratio to show its variation in the Si-containing matrix.

Fourier-transform infrared spectroscopy (FT-IR) was carried out to characterize the coatings. FT-IR spectra (32 scans per analysis at a 4 cm^−1^ resolution) were obtained in transmission mode with a Vertex 70v Bruker spectrometer. The spectrometer was evacuated to less than 150 Pa for 10 min before each acquisition. The spectra were normalized by the maximum intensity of the Si-O-Si stretching band at 1033 cm^−1^.

An X-ray photoelectron spectroscopy (XPS) analysis was carried out to assess the surface chemistry of the coatings with a PHI-5600 VersaProbe spectrometer equipped with a monochromatic Al X-ray source operating at 150 W. Wide-scan and high-resolution C 1s, O1s, Ti 2p, and Si2p spectra were acquired at 115 and 23.5 eV pass energies, respectively. A flood gun (18 mA emission current and 40 eV electron energy) was used for the charge compensation of the samples. The hydrocarbon component of the C 1s spectra was set at 285.0 ± 0.2 eV as a reference for the binding energy scale. The spectral data were processed with MultiPack software version 9.5.

### 2.4. Photocatalytic Activity Evaluation

The photocatalytic activity of the prepared coatings was evaluated by spectrophotometrically monitoring the discoloration of a solution of methylene blue (MB), an organic dye that was selected as target model molecule, assisted by the TiO_2_ nanocomposite-containing coating deposited onto the stainless-steel woven mesh. After the deposition of the nanocomposite film, a 3 × 1 cm^2^ sample of the coated mesh was placed in a quartz cuvette (1 × 1 × 4 cm^3^) containing 3.5 mL of an aqueous solution of MB (10^−5^ M) kept under stirring. A conditioning step was performed in the dark for a 15 min. Then, the system was irradiated with UV light. For this purpose, a UV fluorescent mercury vapor lamp (200 W, λ > 250 nm, light flux 1.2 W/cm^2^) was used, which was equipped with a neutral density filter to reduce the light flux to 0.07 W/cm^2^, as determined with a radiometer (Delta Ohm Data Logger 9721).

At defined time intervals, the absorption spectrum of the solution was recorded, and the absorbance at 665 nm was measured, corresponding to a characteristic peak of MB. The decrease in the intensity of the absorption peak of MB, and hence its discoloration, was monitored over time under the assumption that such a discoloration was accounted for by the degradation of the molecule [34]. The following equation was applied:

$$\%\mathrm{Degradation}\;\mathrm{of}\;\mathrm{MB}=\left[100-\left(\frac{{\mathrm{Abs}}_{t}*100}{{\mathrm{Abs}}_{t_{0}}}\right)\right]$$

where Abs_t_ is the value measured at a given time and Abs_t0_ is the absorbance measured before the exposure to UV. The values were recorded at different exposure times up to 90 min. UV–Vis absorption spectra were recorded with a UV–Vis–NIR Cary 5 (Varian) spectrophotometer.

The absorbance values from the first 15 min were used to calculate the kinetic constant (k) as the slope of the linear fit from the graph with ln(C_0_/C_t_) on the *y*-axis and t on the *x*-axis. The R^2^ of the linear fit is also reported.

## 3. Results and Discussion

Different sets of experiments were performed to investigate the effects of the experimental parameters on the characteristics of the nanocomposite coatings and their photocatalytic performance in order to elucidate the structure–function relationship in the final prepared coatings.

In particular, the effects of different deposition times and the postdeposition O_2_ plasma treatment on the morphological and chemical characteristics and photocatalytic performance of the coatings were investigated. A summary of the experimental parameters used for the preparation of the samples is reported in Table 1.

### 3.1. Effect of the Deposition Time

**Figure 1:**
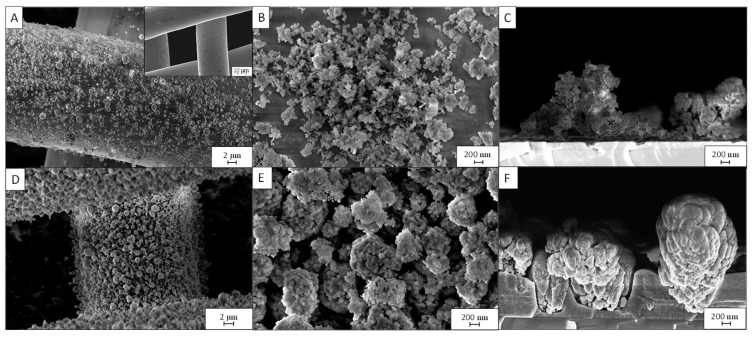
SEM micrographs of the *PD3m* sample at different magnifications: (**A**) top view 5kx and, in the inset, the bare woven stainless-steel mesh; (**B**) top view 50kx; (**C**) cross section 50kx. The *PD20m* sample at different magnifications: (**D**) top view 5kx, (**E**) top view 50kx, (**F**) cross section 50kx. Cross section from samples deposited onto c-silicon.

The *PD3m* and *PD20m* coatings, obtained at two different deposition times, namely 3 and 20 min, respectively, were investigated by SEM, and the obtained micrographs are reported in Figure 1, together with those of the bare woven stainless-steel mesh, before deposition, for comparison.

In Figure 1, for the *PD3m* sample, micrometric and submicrometric aggregates can be observed, superimposed onto a more uniform film that coats the mesh. The morphology of the structures on the surface is consistent with that of TiO_2_ NP aggregates, and they appear to be partially embedded in the hybrid host matrix formed by the plasma process starting from HMDSO and IPA. Indeed, the applied plasma process is expected to activate IPA and HMDSO molecules so as to generate the precursors of plasma polymerization that finally crosslink to form the matrix entrapping the TiO_2_ NPs.

The SEM micrographs of the *PD20m* sample suggest the presence of a thicker coating and likely a higher content of TiO_2_ at the surface, where aggregates are more evident and appear to be only partially embedded in the polymer matrix and still uniformly distributed onto the whole surface of the mesh.

In both samples, the presence of exposed TiO_2_ NPs that are not completely sunk into the matrix can be reasonably expected to beneficially affect the photocatalytic performance of the nanocomposite coatings, as it ensures the accessibility of the photocatalytic sites to the pollutant species dispersed in the water for their effective photocatalytic degradation [8,20]. The SEM images acquired at a higher magnification show exposed isolated NPs with diameters in the range reported for the TiO_2_ P25 powder (10–50 nm) and some small aggregates covered by a polymer layer that slightly increases their diameters (see Supplementary Materials, Figure S4).

The thickness of the coating, as measured by a profilometer, ranged from 234 ± 9 nm for *PD3m* to 1940 ± 90 nm for *PD20m*.

The XPS analysis performed on the *PD3m* and *PD20m* coatings resulted in the elemental composition, expressed as the atomic concentration, reported in Table 2. Passing from the *PD3m* to the *PD20m* sample, an increase in the amount of Ti was observed, along with much higher O and Si contents and a relevant decrease in C, thus indicating that during the deposition process the film composition changed.

In fact, such evidence is somehow unexpected since the deposition time is an experimental parameter that is typically found to scarcely affect the characteristics of plasma-assisted film, considering that the sample temperature does not increase substantially, which is the case for these experiments. A possible explanation for the observed evolution in the coating composition with deposition time can be found in the photocatalytic activity of the TiO_2_ NPs, which could play a significant role in such a phenomenon. UV light produced in the plasma, irradiating the TiO_2_ in the sample, can induce the photocatalytically assisted degradation of the organic component of the hybrid host matrix. Such a hypothesis is based on the photogeneration of oxygen-containing radicals in the plasma phase, which is reasonable due to the presence of water vapor and O_2_ desorbing from the reactor walls and electrodes. On the other hand, the decomposition of HMDSO and IPA in the plasma could also, in principle, lead to the formation of oxygen-containing species that could be involved in a self-photocatalytic oxidation process during the plasma deposition. Such a phenomenon has, to the best of our knowledge, not been attested so far. Therefore, further investigation is necessary to thoroughly rationalize the process.

**Figure 2:**
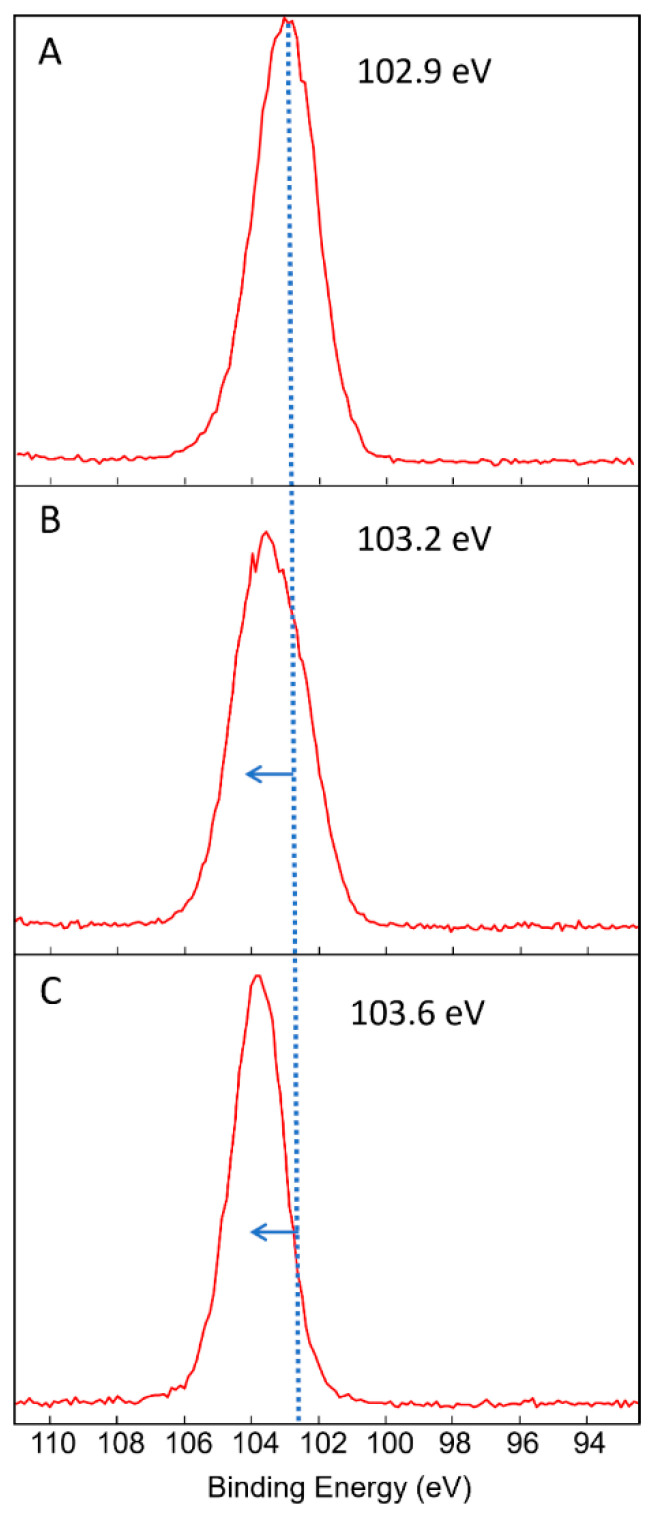
Si2p peak acquired by XPS peak analyses of the *PD3m* (**A**), *PD20m* (**B**), and *PD3m-P* (**C**) coatings. A shift toward a higher binding energy can be seen for the *PD20m* and *PD3m-P* samples compared to *PD3m*.

The XPS peak analyses (Figure 2) show a shift in the Si2p peak from 102.9 eV (*PD3m*) to 103.2 eV (*PD20m*), highlighting the oxidation of Si from the organosilicon state (Si-C) to the SiO_2_-like state, thus suggesting that the removal of the organic component is related to the formation of new Si-O bonds and the removal of organic moieties.

### 3.2. Effect of the Postdeposition Plasma Treatment

A postdeposition treatment was performed by exposing the coatings prepared with both short (*PD3m*) and long (*PD20m*) deposition times to a low-pressure O_2_ plasma, and the *PD3m-P* and the *PD20m-P* samples, respectively, were obtained. Indeed, the reactive oxygen atoms originating in the plasma were expected to oxidize the matrix, thus reducing the organic component therein and leaving SiO_x_ as the main inorganic domain. Such a treatment, therefore, resulted in (i) an increase in the exposed fraction of the TiO_2_ NPs that were originally partially embedded in the matrix and (ii) an increase in the inorganic component of the organosilicon matrix, which was a more inorganic coating that was chemically resistant and was able to sustain any possible photocatalytic action of the inorganic photocatalytic fillers and thus was also less prone to the electron-scavenging effect.

To evaluate the effect of the postdeposition plasma treatment, a comparison was carried out between the *PD3m* and *PD3m-P* coatings.

The elemental compositions of *PD3m* and *PD3m-P* coatings were evaluated by XPS and are reported in Table 2.

The postdeposition treatment was found to induce a reduction in C and increases in the O and Si atomic percentages with respect to the starting *PD3m* coating. Such evidence suggests that the treatment was responsible for the removal of part of the organic component from the hybrid host matrix that forms the coating, along with of a general oxidation process of the matrix. The results of the XPS peak analyses (Figure 2) show that the Si2p peak shifted from 102.9 eV (*PD3m*) to 103.6 eV (*PD3m-P*), highlighting the oxidation of Si from the organosilicon state to the SiO_2_-like state, as the removal of the organic component of the matrix resulted in the formation of additional Si-O bonds. On the other hand, in the O1s signal, a component at 530 eV, which is typically ascribed to Ti-O bonds [35,36], appeared more intense in the *PD3m-P* coating beside the dominant and broad contribution at a high binding energy attributed to the O-C and O-Si bonds.

An EDS analysis was also performed in order to investigate the coating at a sampling depth higher than that probed by XPS, and the results are reported in Table 3 in terms of the Ti/Fe and Ti/Si atomic concentration ratios. The Ti/Fe ratio remained fairly constant, while the Ti/Si ratio increased from 0.21 to 0.49 for *PD3m-P*. Considering that the presence of Fe can be ascribed to composition of the steel mesh, it can be inferred that the total amount of Ti on the substrate remained constant while the coating was depleted of the host matrix component, as testified by the Ti/Si increase. Therefore, the postdeposition plasma treatment significantly affected the nature of the coating, modifying the host matrix composition and increasing the relative amount of TiO_2_ on the surface.

**Figure 3:**
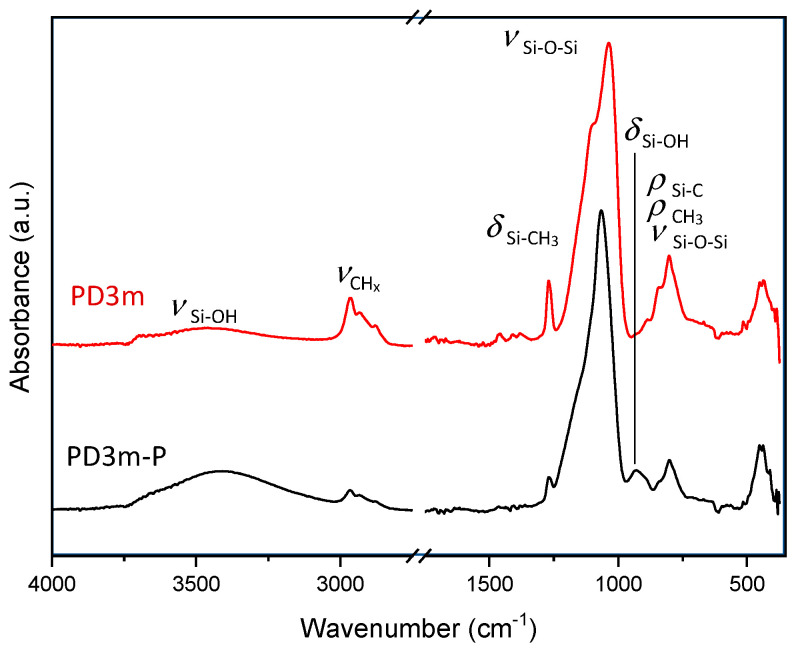
FT-IR spectra of *PD3m* and *PD3m-P* coatings.

The FT-IR spectra (Figure 3) of the *PD3m* and *PD3m-P* samples show, after the postdeposition plasma treatment, reductions in the intensity of the signals at 2970–2878 cm^−1^ (stretching of -CH_3_), 1266 cm^−1^ (bending of Si-CH_3_), and 805–887 cm^−1^ (Si-C and -CH_3_ bending), related to the hydrocarbon component. On the other hand, increases in the intensity of the signals ascribed to Si-OH stretching in the 3000–3700 cm^−1^ region and the bending of Si-OH at 933 cm^−1^ can be observed. The intense peak at 1033 cm^−1^, distinctive of the stretching of the Si-O-Si covalent bond, was found to become sharper after the postdeposition treatment and to shift towards higher wavenumbers, moving from 1033 cm^−1^ for the *PD3m* sample to 1067 cm^−1^ for the *PD3m-P* sample. This behavior typically indicates a transition towards a SiO_2_-like structure, according to literature [37]. Therefore, the Si-O-Si peak analysis and the reduction in the carbon-containing moieties concur in demonstrating the more inorganic nature of the coating after the plasma treatment.

The thickness of the coatings was measured by using a profilometer, resulting in measurements of 190 ± 10 nm and 1830 ± 30 nm for the *PD3m-P* and the *PD20m-P* coatings, respectively. These values were lower than those measured for their untreated counterparts (234 ± 9 nm for *PD3m* to 1940 ± 90 nm for *PD20m*), thus further supporting the thesis of a slight thinning of the coating due to the removal of the carbonaceous component.

### 3.3. Photocatalytic Activity of the Nanocomposite Coatings

The photocatalytic performances of the prepared coatings were evaluated by monitoring the degradation of MB upon UV light irradiation.

**Figure 4:**
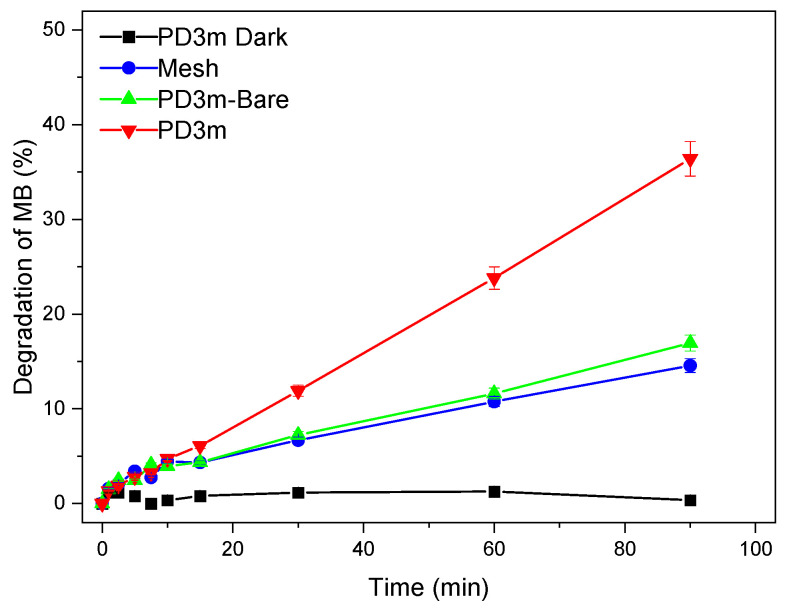
MB degradation experiments were monitored upon UV irradiation with the naked mesh (Mesh), the mesh coated with the organic matrix of the coating (*PD3m-bare*), and the *PD3m* coating (*PD3m*). For comparison, the experiment was also carried out while exposing the 3m coating to the dye solution in the dark (*PD3m* Dark). The experimental data are reported as the mean values of three replicates ± standard deviations.

The degradation experiments with the *PD3m* coating resulted, after 90 min, in 36% degradation, while for the *PD3m-bare* sample there was only 15% degradation (Figure 4). In the case of the *PD3m-bare* sample, the degradation was clearly ascribed to the UV-irradiation-induced photolysis of the MB. In fact, the same degradation extent was observed by irradiating a bare stainless-steel mesh, which was also in this case ascribable only to direct photolysis. Moreover, the *PD3m* coating exposed to the solution in the dark demonstrated ineffective degradation of MB, thus confirming the photoinduced nature of the degradation process. Moreover, since the degradation extent was found to be comparable for *PD3m-bare* and the bare mesh, the polymer host matrix can be safely considered photocatalytically ineffective and unable to specifically adsorb the MB present in the solution.

**Figure 5:**
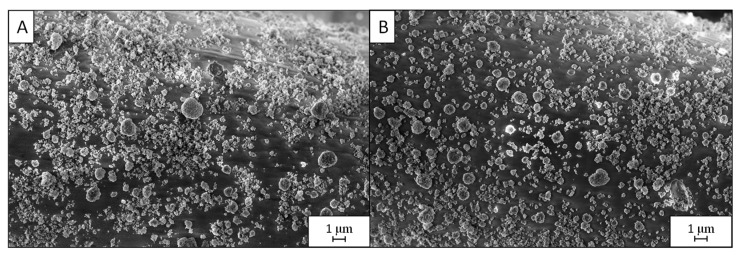
SEM micrographs of *PD3m* before (**A**) and after (**B**) the photocatalytic process (top view at 10kx magnification).

The *PD3m* coating was investigated by SEM before and after the photocatalytic experiment in order to assess a possible modification in the morphology due to the immersion in an aqueous solution and exposure to UV light. The results, reported in Figure 5, show that the coating morphology was preserved after 90 min of UV irradiation while immersed in the aqueous MB solution.

The EDS data, reported in Table 4, indicate that only a slight reduction in the Ti content could be detected after the experiment. Overall, these results confirm the reasonable coating stability during the photocatalytic experiment.

**Figure 6:**
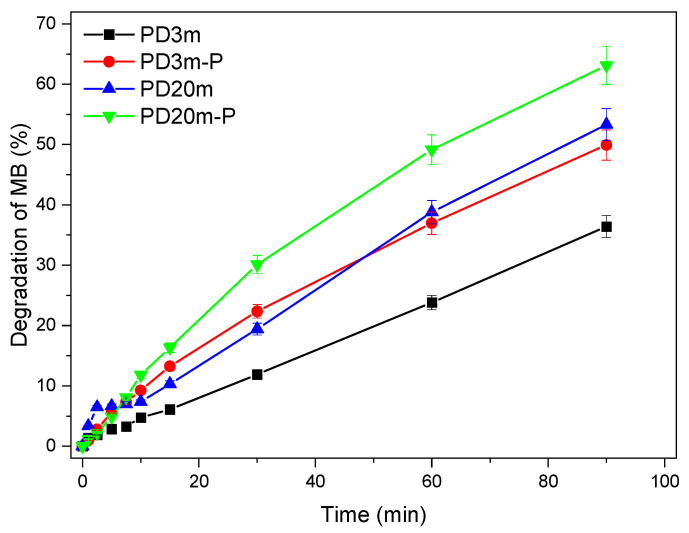
MB degradation assisted by the *PD3m*, *PD3m-P*, *PD20m*, and *PD20m-P* coatings. The experiments were carried out under UV light irradiation. The MB concentration was evaluated by monitoring the absorbance intensity at 665 nm. The experimental data are reported as the mean values of three replicates ± standard deviations.

The photocatalytic efficiencies of all the TiO_2_-containing coatings in degrading MB are reported in Figure 6, and after 90 min of UV light exposure, 36% degradation was reached for *PD3m*, 50% degradation was reached for *PD3m-P*, 53% degradation was reached for *PD20m,* and 63% degradation was reached for *PD20m-P*. The absorbance spectra of the MB solution at different times of exposure to UV light and contact with *PD20m-P* are reported as an example in the Supplementary Materials (Figure S5). It can be observed that the coatings obtained with longer deposition times (*PD20m* and *PD20m-P*) presented photocatalytic activities higher than those shown by *PD3m* and *PD3m-P*. Moreover, the postdeposition plasma treatment, in both cases, resulted in a further enhancement of the photocatalytic performance, which was more relevant for the *PD3m* coating. The values of the degradation percentage achieved at 90 min of irradiation are reported in Table 5.

The apparent rate constants (k) of the MB degradation experiments were calculated from the degradation data collected in the first 15 min as the slope of the straight line obtained when reporting ln(C_0_/C) vs. reaction time. The resulting k and the R^2^ of the linear fitting used for the elaboration are reported in Table 5. Interestingly, higher degradation values correspond to a higher k and hence a faster degradation reaction. These data also point out that similar photocatalytic activities could be obtained by increasing the deposition time or by applying a postdeposition plasma treatment.

**Figure 7:**
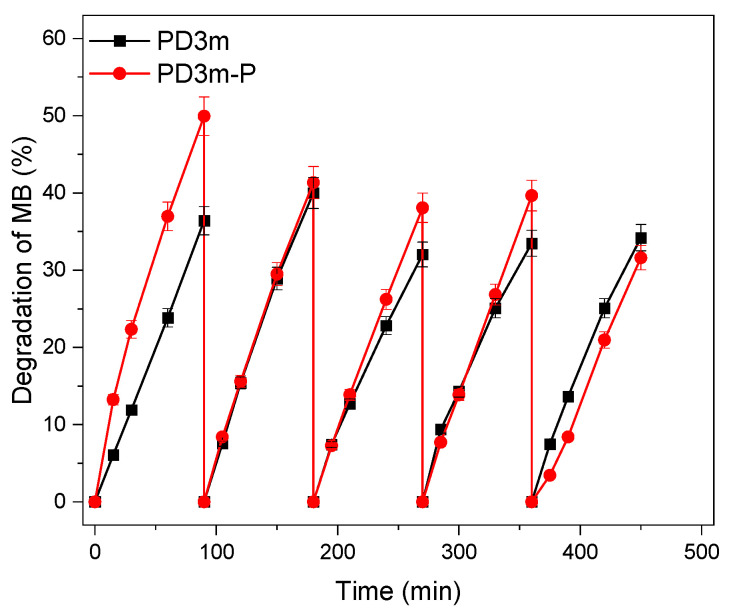
Photocatalytic performance of *PD3m* and *PD3m-P* in the MB degradation experiment, measured over five cycles.

The reusability of the *PD3m* and *PD3m-P* coatings was tested for five cycles of MB degradation, and the results are illustrated in Figure 7.

**Figure 8:**
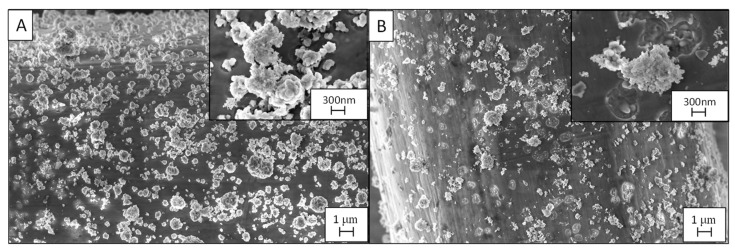
SEM micrographs of *PD3m-P* coating before (**A**) and after (**B**) the five use cycles; the images were acquired at 10kx magnification, and the insets were acquired at 50kx.

The *PD3m* coating presented good stability over the utilization, retaining a steady degradation value that remained higher than 33%. For *PD3m-P,* the postdeposition plasma treatment was demonstrated to significantly improve the photocatalytic activity of the coating, with a 50% degradation value in the first experiment (Figure 6) and with constant values similar to *PD3m* in the subsequent experiments. The SEM investigation reported in Figure 8 shows that the morphology of the *PD3m-P* sample obtained after the postdeposition plasma treatment (Figure 8A) did not differ significantly from the *PD3m* coating. The SEM analysis also highlighted the occurrence in the *PD3m-P* sample of holes on the surface after five experiments, probably due to the detachment of material from the coating surface (Figure 8B).

The EDS data for *PD3m-P* point out a reduction in the Ti/Fe ratio from 0.09 ± 0.01 to 0.04 ± 0.01 after five subsequent experiments, but the Ti/Si ratio remained constant (0.54 ± 0.03 from 0.49 ± 0.02). This could be interpreted as a reduction in the total amount of Ti on the surface, concomitant to a loss of the matrix component, that led to a constant Ti/Si. The SEM images (Figure 8B) indicate a loss of material from the coating, but, on the other hand, the EDS data show a constant Ti/Si ratio, suggesting that a thinning of the coating in its entirety has occurred, rather than the detachment of TiO_2_ aggregates.

This evidence could account for the trend observed in the *PD3m-P* photocatalytic activity reported in Figure 7. In fact, the performance, which was high in the first experiment, was then reduced in the second one, probably due to the removal of the less firmly anchored material, and reached a steady value in the following experiments.

**Figure 9:**
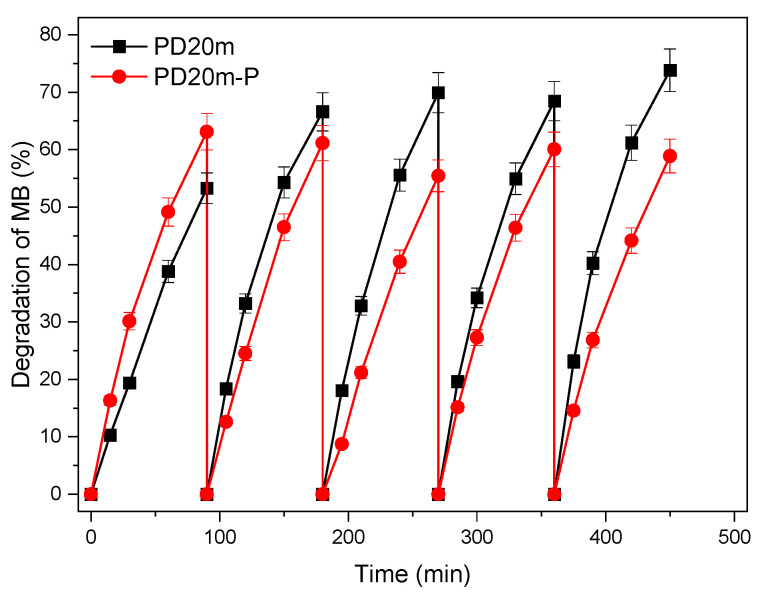
Photocatalytic performance of the *PD20m* and *PD20m-P* coatings in the MB degradation experiment, measured over five cycles.

The photocatalytic activities of the *PD20m* and *PD20m-P* coatings were also evaluated over five repeated experiments, and the results are reported in Figure 9. In the first experiment, *PD20m-P* showed an activity higher than *PD20m*. However, in the following experiments, the *PD20m-P* activity remained stable around a value of 60% MB degradation. On the other hand, the activity of the *PD20m* coating increased in the first three experiments, then remained stable around the value of 70% MB degradation. The behavior was very similar to that observed for the *PD3m* and *PD3m-P* samples and reported in Figure 7. In this case, the enhancement of the photocatalytic activity due to the postdeposition plasma treatment recorded in the first experiment was, however, somehow not retained after repeated experiments. However, even after five experiments, both prepared coatings still exhibited activities higher than those found for *PD3m* and *PD3m-P*, without any evident loss of performance.

**Figure 10:**
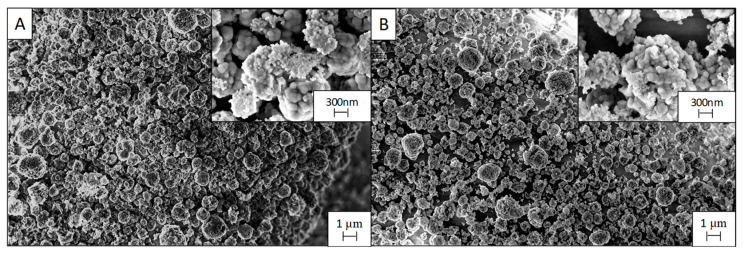
SEM images of the *PD20-P* coating before (**A**) and after (**B**) the five use cycles; the images were acquired at 10kx magnification, while the insets were acquired at 50kx.

The SEM micrographs (Figure 10) of the *PD20m-P* coating and the *PD20m* sample do not differ significantly, thus highlighting that the postdeposition plasma treatment did not affect the morphology of the coating. In this case, no changes or aggregate detachment were visible after the five repeated experiments, in contrast with for the observations of the *PD3m-P* coating (Figure 10B).

Moreover, from a chemical point of view, the Ti/Fe and Ti/Si ratios from the EDS only slightly changed after the entire recycling process. The stability of the *PD20m-P* coating was higher than that exhibited by *PD3m-P*. Such a finding can be explained by the more inorganic nature of its host matrix, as highlighted by the XPS elemental composition (Table 2) and the peak analysis. In addition, the *PD20m* coating was thicker than the *PD3m* coating.

The postdeposition plasma treatment modified the *PD3m* coating by oxidizing the carbonaceous compound of the matrix. It seemed to alter the *PD20m* coating much less since its matrix was already characterized by a quite oxidized nature. In this case, the plasma treatment could have resulted in an excessive weakening of the *PD3m* coating, while the already quite resistant *PD20m* coating was not significantly affected. The stronger effect of the plasma treatment on *PD3m* than on *PD20m* is consistent with the observed enhancement of the photocatalytic activity compared to that of the *PD20m* coating.

## 4. Conclusions

This work presents the deposition of photocatalytic composite coatings containing TiO_2_ on stainless-steel mesh for the degradation of organic pollutants. The morphological and chemical characteristics and the photocatalytic activities of different nanostructured TiO_2_-containing coatings deposited by means of aerosol-assisted atmospheric-pressure plasma were investigated.

Thicker coatings, obtained with longer deposition times, were characterized by a reduced organic content and a relevant SiO_x_-like character at the surface, while the bulk of the coating did not show any significant change in chemistry. Their photocatalytic activities were found, in any case, to be higher than those recorded for the coatings obtained with shorter deposition times.

The postdeposition plasma treatment was found to reduce the thickness of the coating and, concomitantly, their organic content, both at the surface and in the bulk, resulting in an overall more oxidized, more inorganic, and SiO_x_-like film that still retained the original TiO_2_ content. Remarkably, such a postdeposition treatment resulted in an increase in the photocatalytic activity.

In general, all the prepared coatings tested in reuse experiments mainly retained their photocatalytic activities, with only slight differences observed, which were accounted for by their specific structures and morphologies.

To the best of our knowledge, this is the first report of TiO_2_-containing silicone-like coatings deposited by means of aerosol-assisted atmospheric-pressure plasma that assesses their photocatalytic activity and the effect of a plasma posttreatment for increasing their efficiency.

The proposed deposition technique resulted in a manageable and flexible tool for immobilizing a nanostructured photocatalyst onto a technologically relevant substrate, such as stainless-steel mesh, providing effective photocatalytic coatings that are stable over time and are suited for the application of the photocatalytic step for organic contaminant degradation in water treatment but are also amenable to being integrated in other state-of-the-art technologies, such as food packaging, antimicrobial surfaces, and functional textiles.

## Figures and Tables

**Table 1:** Summary of the experimental parameters of the coating preparations (24 kHz/5 kV_pp_).

Sample	Deposition Time (min)	Aerosol Composition	Post Deposition O_2_ Plasma Treatment
*PD3M*	3	TiO_2_ (10 mg/mL) HMDSO/IPA (10/90 *v*/*v*)	No
*PD20M*	20	TiO_2_ (10 mg/mL) HMDSO/IPA (10/90 *v*/*v*)	No
*PD3M-P*	3	TiO_2_ (10 mg/mL) HMDSO/IPA (10/90 *v*/*v*)	Yes
*PD20M-P*	20	TiO_2_ (10 mg/mL) HMDSO/IPA (10/90 *v*/*v*)	Yes
*PD3M-BARE*	3	HMDSO/IPA (10/90 *v*/*v*)	No

**Table 2:** XPS elemental compositions of *PD3m*, *PD20m*, and *PD3m-P* coatings.

Elements	*PD3m (%)*	*PD20m (%)*	*PD3m-P (%)*
C	53 ± 3	8.8 ± 0.4	8.0 ± 0.4
O	29 ± 1	63 ± 3	67 ± 3
Si	17.3 ± 0.9	27 ± 1	24 ± 1
Ti	0.4 ± 0.1	1.2 ± 0.1	1.4 ± 0.1

**Table 3:** Ti/Fe and Ti/Si atomic concentration ratios obtained by EDS of the *PD3m* and *PD3m-P* coatings.

Sample	Ti/Fe	Ti/Si
*PD3m*	0.10 ± 0.03	0.21 ± 0.02
*PD3m-P*	0.09 ± 0.03	0.49 ± 0.03

**Table 4:** Ratios between the atomic concentration percentages measured by EDS on the *PD3m* coatings before and after the photocatalytic process.

Sample	Ti/Fe	Ti/Si
*PD3m before*	0.10 ± 0.03	0.21 ± 0.02
*PD3m after*	0.07 ± 0.03	0.18 ± 0.03

**Table 5:** The kinetic constants, R^2^, and degradation achieved after 90 min of UV irradiation of the different coatings.

Sample	k (min^−1^)	R^2^	Degradation after 90 min
*PD3m*	0.0039	0.98	36±2%
*PD20m*	0.0087	0.98	53±3%
*PD3m-P*	0.0094	0.99	50±2%
*PD20m-P*	0.0122	0.99	63±3%

## Data Availability

Data is contained within the article or supplementary material.

## Supplementary Materials

The following supporting information can be downloaded at: https://www.mdpi.com/article/10.3390/nano12213758/s1, Figure S1: Images of TiO_2_ P25 (Evonik) acquired by (A) scanning electron microscopy (SEM) at 300kx magnification and (B) transmission electron microscopy (TEM); Figure S2: Scheme of the dielectric barrier discharge (DBD) plasma reactor used for the plasma deposition; Figure S3: Scheme of the low-pressure plasma reactor used for the plasma postdeposition treatment; Figure S4: SEM image at 100kx magnification of *PD20m* coating; Figure S5: Absorbance spectra for 10^−5^ M methylene blue (MB) over time (90 min in total) of exposure to UV light and contact with *PD20m-P* as a representative evolution.
