# Supplementary material for: Photocatalytic Investigation of Aerosol-Assisted Atmospheric Pressure Plasma Deposited Hybrid TiO2 Containing Nanocomposite Coatings
Source: Nanomaterials (Basel). 2022 Oct 26;12(21):3758. doi: 10.3390/nano12213758 (PMC9653690; doi:10.3390/nano12213758)
Supplement: Supplementary file 1 [file nanomaterials-12-03758-s001.zip › nanomaterials-1962788-supplementary.pdf]

Supplementary materials

Photocatalytic Investigation of Aerosol-Assisted Atmospheric Pressure Plasma Deposited Hybrid TiO_2 Containing Nanocomposite Coatings

Chiara Lo Porto [1], Massimo Dell'Edera [1], Ilaria De Pasquale [1], Antonella Milella [2,3], Francesco Fracassi [2,3], Maria Lucia Curri [1,2,*], Roberto Comparelli [1,*] and Fabio Palumbo [3,*]

[1] CNR-IPCF, Istituto per i Processi Chimico-Fisici, S.S. Bari, c/o Dip. Chimica Via Orabona 4, 70126 Bari, Italy

[2] Dipartimento di Chimica, Università degli Studi di Bari Aldo Moro, Via Orabona 4, 70126 Bari, Italy

[3] CNR-NANOTEC, c/o Dip. Chimica Via Orabona 4, 70126 Bari, Italy

* Correspondence: marialucia.curri@uniba.it (M.L.C.); roberto.comparelli@cnr.it (R.C.); fabio.palumbo@cnr.it (F.P.)

Nanoparticles TiO_2 P25 characterization

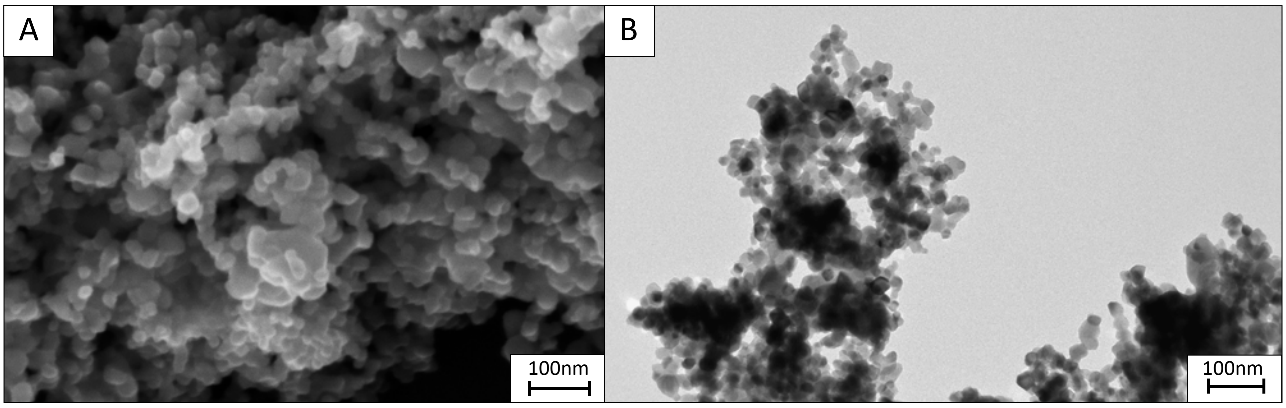

Figure S1 Images of TiO_2 P25 (Evonik) acquired by A) scanning electron microscopy (SEM) at 300kx magnification and B) transmission electron microscopy (TEM)

Transmission electron microscopy (TEM) analysis was performed by a JEOL JEM-1011 microscope operating at 100 kV. The TEM samples were prepared by casting a drop of TiO_2 methanol solution onto a carbon hollowed TEM grid.

Plasma reactors

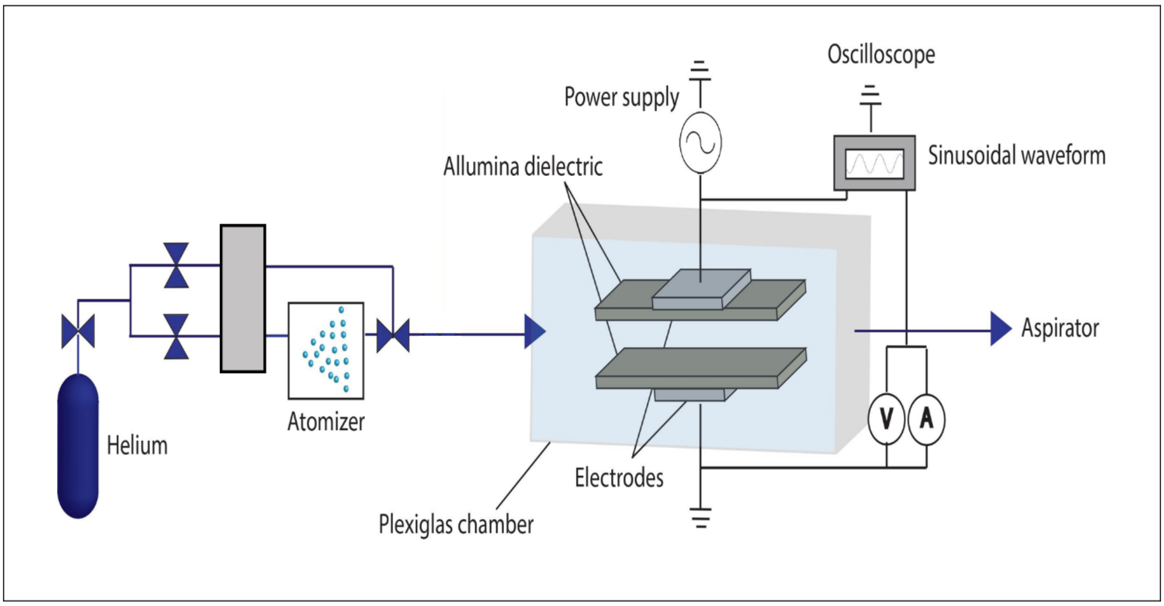

Figure S2 Scheme of the Dielectric Barrier Discharge (DBD) plasma reactor used for the plasma deposition. [1]

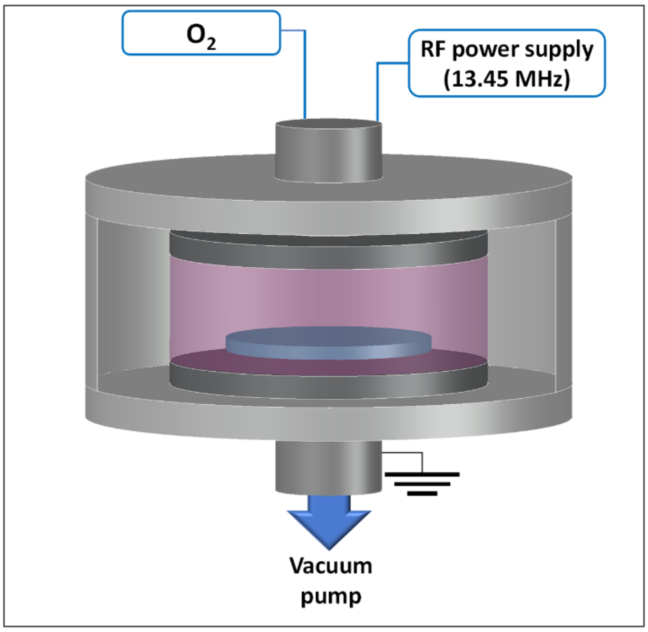

Figure S3 Scheme of the low pressure plasma reactor used for the plasma post-deposition treatment.[2]

Coating characterization

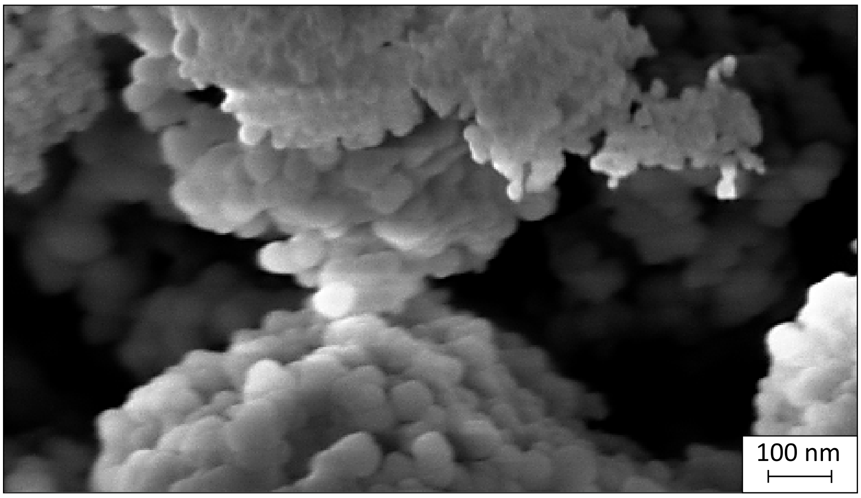

Figure S4 SEM image at 100kx magnification of PD20m coating.

Test of the photocatalytic activity of the prepared coatings

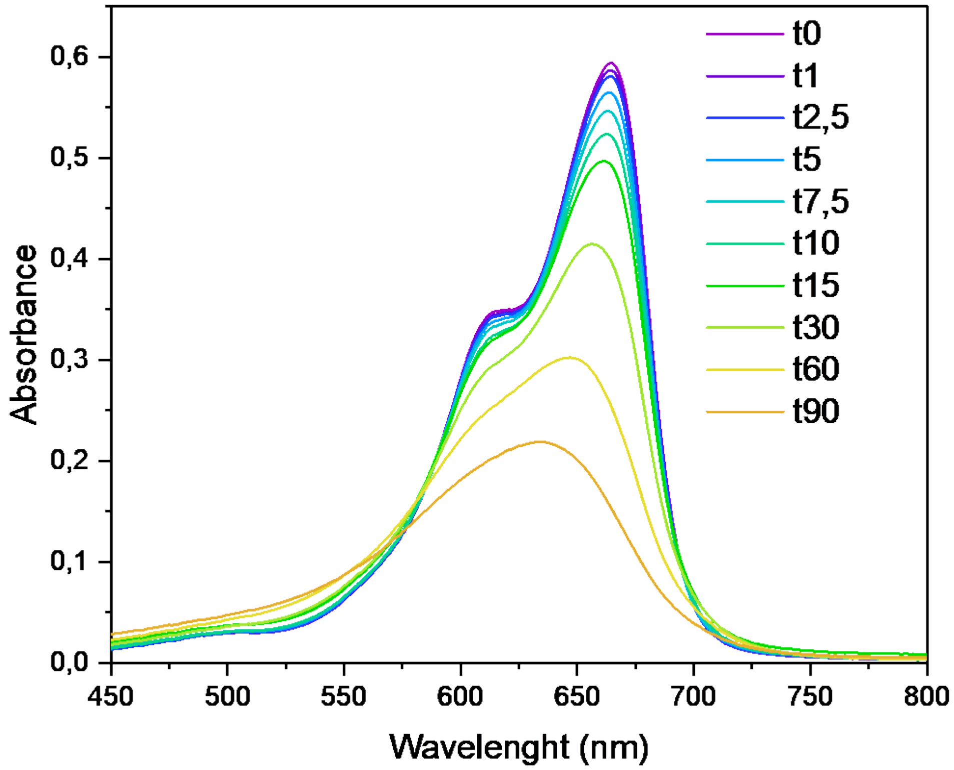

Figure S5 Absorbance spectra for 10^{-5}M methylene blue (MB) over time (90 minutes in total) of exposure to UV light and contact with PD20m-P as a representative time evolution.

References

1. Lo Porto, C.; Palumbo, F.; Buxadera-Palomero, J.; Canal, C.; Jelinek, P.; Zajickova, L.; Favia, P. On the Plasma Deposition of Vancomycin-Containing Nano-Capsules for Drug-Delivery Applications. *Plasma Process. Polym.* **2018**, *15* (5), 1700232. https://doi.org/10.1002/ppap.201700232.
2. Lo Porto, C.; Palumbo, F.; Somma, S.; Masiello, M.; Moretti, A.; Fracassi, F.; Favia, P. Plasma-Assisted Deposition of Fungicide Containing Coatings for Encapsulation and Protection of Maize Seeds. *Plasma Process. Polym.* **2019**, *16* (6), 1900022. https://doi.org/10.1002/ppap.201900022.
